# A Frequency-Domain Multipath Parameter Estimation and Mitigation Method for BOC-Modulated GNSS Signals

**DOI:** 10.3390/s18030721

**Published:** 2018-02-28

**Authors:** Chao Sun, Hongbo Zhao, Wenquan Feng, Songlin Du

**Affiliations:** Electronic and information Engineering Department, Beihang University, 100191 Beijing, China; sunchao@buaa.edu.cn (C.S.); buaafwq@buaa.edu.cn (W.F.); hello508@buaa.edu.cn (S.D.)

**Keywords:** frequency domain analysis, multipath channels, parameter estimation, satellite navigation systems, binary offset carrier (BOC) modulation

## Abstract

As multipath is one of the dominating error sources for high accuracy Global Navigation Satellite System (GNSS) applications, multipath mitigation approaches are employed to minimize this hazardous error in receivers. Binary offset carrier modulation (BOC), as a modernized signal structure, is adopted to achieve significant enhancement. However, because of its multi-peak autocorrelation function, conventional multipath mitigation techniques for binary phase shift keying (BPSK) signal would not be optimal. Currently, non-parametric and parametric approaches have been studied specifically aiming at multipath mitigation for BOC signals. Non-parametric techniques, such as Code Correlation Reference Waveforms (CCRW), usually have good feasibility with simple structures, but suffer from low universal applicability for different BOC signals. Parametric approaches can thoroughly eliminate multipath error by estimating multipath parameters. The problems with this category are at the high computation complexity and vulnerability to the noise. To tackle the problem, we present a practical parametric multipath estimation method in the frequency domain for BOC signals. The received signal is transferred to the frequency domain to separate out the multipath channel transfer function for multipath parameter estimation. During this process, we take the operations of segmentation and averaging to reduce both noise effect and computational load. The performance of the proposed method is evaluated and compared with the previous work in three scenarios. Results indicate that the proposed averaging-Fast Fourier Transform (averaging-FFT) method achieves good robustness in severe multipath environments with lower computational load for both low-order and high-order BOC signals.

## 1. Introduction

Multipath is one of the dominating error sources in high precision global navigation satellite system (GNSS) applications. It depends on the local environment and cannot be removed by using differential corrections. This introduces a considerable error to the pseudorange observation estimation. To minimize the impact of multipath, multipath mitigation approaches have been widely studied and employed in the user receivers.

Generally, multipath mitigation techniques are classified into three main categories [1,2]: (1) pre-receiver mitigation techniques are applied prior to the GNSS signal entering the receiver trying to minimize the received multipath power; (2) receiver signal processing techniques work within the code and carrier tracking loops to compensate the effect of multipath; (3) post-processing techniques occur after the GNSS pseudo-range measurements have been produced. For this paper, the second category is the subject.

Along with the implementation of the GPS modernization program and the development of other global navigation systems, such as Galileo, BeiDou, and GLONASS, binary offset carrier (BOC) and its derivative signals are designed and adopted to achieve significant enhancement but also introduce challenges. Conventional multipath mitigation techniques were generally designed for BPSK-modulated signal whose autocorrelation function is a triangle with single peak, such as GPS coarse-acquisition (C/A) code, whereas for BOC-modulated signals, because of subcarrier, the autocorrelation function has multi-peaks. They will result in frequent false locking and large ranging errors under a severe multipath circumstance [3]. Therefore, developing reliable and effective multipath mitigation techniques for BOC signals, especially high-order BOC signals, becomes a pressing need [4].

Currently, some multipath mitigation approaches have been presented specifically aiming at BOC signals. They can be further classified into two categories: non-parametric techniques and parametric techniques [5].

### 1.1. Non-Parametric Techniques for BOC Signals

Typically, the most widely used multipath mitigation techniques are non-parametric techniques. They avoid false locking on the secondary peaks of BOC signals by using original code delay discriminators or designing particular local reference code waveforms for correlation.

Code Correlation Reference Waveforms (CCRW) [6] is a representative of this type. In a broad sense, shaping correlator [7], BOC-Gated-PRN [8,9,10], gating functions (GF) [1] and even Narrow Correlator^TM^ [11] and double-delta correlation variants (high resolution correlator (HRC), strobe correlator, etc.) [12,13,14,15] all belong to this family. For example, if the incoming signal is correlated with a local specially designed CCRW, we can get a discriminator curve equivalent to that of the Narrow Correlator ^TM^. Nevertheless, these techniques have to carefully design different local reference PRN sequences for each specific type of BOC signals, which shows low generality [16]. For example, the shaping correlator method, developed for BOC(1,1) modulation, is not applicable to other high-order BOC signals [7]. BOC-Gated-PRN is just effective on BOC(*n*,*n*) signals and MBOC signal, but not effective for other signals [8,9]. Another kind of CCRW, named as gating functions, can be used on BOC(*n*,*n*) and BOC(2*n*,*n*) signals, but it still cannot process arbitrary BOC(*m*,*n*) signals, such as BOC(14,2) [1]. Besides, it is also worth to mention some other well-known limitations, namely degraded carrier-to-noise ratio and poor tracking dynamic performance, which significantly limit the applicability of such methods [7].

### 1.2. Parametric Techniques for BOC Signals

The second class of multipath mitigation techniques is the parametric ones. Theoretically, parametric techniques can thoroughly eliminate the impact of multipath signals by estimating the characteristics (time delay, amplitude and relative carrier phase) of both the line of sight (LOS) and the reflected signals.

The maximum likelihood approach, such as multipath estimating delay locked loop (MEDLL) [17], is among the most popular parametric techniques. Techniques of this type outperform others since they have the potential to sweep away multipath signals more thoroughly but at the cost of relatively high computational load. Besides, they also need a-priori assumptions about the number of extra paths. When this priori information is not consistent with the fact, considerable estimation errors may be introduced. So, they cannot work properly when the number of paths is very large or unknown [18].

Another kind of parametric methods is the so-called subspace estimation techniques, namely the multiple signal classification (MUSIC) [19], and its modified method, estimation of signal parameters via rotational invariance technique (ESPRIT) [20,21]. This class of method can estimate not only the characteristics of multipath signals but also the number, so it does not need any a priori information. The weakness of this method lies in its bad capacity of estimating the amplitude of multipath components and high sensitivity to the noise. Besides, because of large-scale matrix transformations and singular value decomposition, its computational burden is usually too high for practical application [18].

Anti-multipath techniques in the frequency domain is also a promising category of parametric methods. It is different from most conventional techniques implemented in the time domain. Reference [22] implemented multipath detection by monitoring the abnormal energy in the frequency domain, but no mitigation is performed. The satellite whose LOS signal is severely affected by reflected paths is excluded from the navigation solution. Actually, it is not a parametric technique. Reference [23] presented a frequency domain processing method by nulling out the multipath frequency spectrum. But, this technique requires satellite elevation and antenna height as a-priori information to estimate the static multipath parameters before multipath mitigation. It may be effective for a fixed receiver, such as the monitoring receivers used in the Wide Area Augmentation System (WAAS), but infeasible for a moving receiver with changing surroundings. Another interesting kind of frequency domain processing method is the FFT-based method presented by [24,25]. Mathematically, the superposition of multipath signals in the time-domain is equivalent to the spectrum multiplication of the line of sight (LOS) and a multipath channel transfer function (CTF) in the frequency-domain. This principle can be employed to achieve anti-multipath purpose in the frequency domain. As this FFT-based method obtains the power spectra by directly taking FFT without statistical averaging, we just call it direct-FFT method in this paper. The direct-FFT method is not sensitive to different modulation types, as well as the number and amplitudes of multipath signals, showing high feasibility for different scenarios. Nevertheless, this method is still suboptimal for its high computation complexity and vulnerability to the noise.

To sum up, though the parametric techniques show better multipath mitigation performance on BOC signals compared with the non-parametric techniques, they are generally limited by the excessive algorithm complexity and sensitivity to the noise. Basically, if we have a carrier to noise ratio (C/N_0_) higher than 40 dB-Hz, we are likely in strong LOS scenario, thus without multipaths [26]. It is important to study such algorithms below 40 dB-Hz. Besides, the adoption and promotion of BOC-modulations also make multipath mitigation tougher. Thus to make the parametric techniques feasible in numerous real applications especially for BOC signals, breakthroughs from two aspects are required, which are improving the robustness to the noise and reducing the computation complexity.

To deal with the above problems, we present a modified frequency-domain technique to estimate multipath estimation parameters method for arbitrary BOC(*m*,*n*) signals which is called averaging-FFT method or aFFT method for simplicity in this paper. By the processes of cross power spectral density (CPSD) estimation and spectrum division, the proposed method can estimate the multipath parameters quantitatively and then mitigate the multipath signals. The principal contribution of this paper is that, compared with other parametric techniques, the proposed method provides better anti-multipath capability with acceptable algorithm complexity, which makes it an interesting candidate for real applications. The performance analysis is implemented on BOC(1,1), BOC(14,2) and BOC(15,2.5) signals. Besides, the performance comparison between the direct-FFT method, MEDLL and CCRW techniques are also provided. Results show that the averaging-FFT method is feasible to perform multipath parameter estimation for BOC(*m*,*n*) signals of arbitrary types with various numbers of multipath signals.

## 2. Signal and Channel Model

Before presenting the proposed method, we first introduce the signal model used in this paper. We begin this section by deriving the expression of multipath CTF which characterizes the multipath channel in the frequency domain, and then briefly introduce the principle of the direct-FFT method.

### 2.1. Multipath Channel Transfer Function

Multipath fading effect can be characterized by four parameters: the number of reflected signals, amplitude, time delay, and phase difference of each path relative to LOS. Assuming the incoming signal is an arbitrary BOC-modulated signal, it can be modeled as:

(1) $$s\left(t\right)=s_{0}\left(t\right)+\sum_{i=1}^{P}s_{i}\left(t\right)=\sum_{i=0}^{P}\alpha_{i}Ac\left(t-\tau_{i}\right)sc\left(t-\tau_{i}\right)\cos\left(w_{0}t+\varphi_{i}\right)$$

where $s_{0}\left(t\right)$ is the LOS, $s_{i}\left(t\right)$ denotes the *i*-th multipath signal, *P* is the total number of multipath signals, *A* is the amplitude of the LOS, c(t) represents the pseudo-random code and $sc\left(t-\tau_{i}\right)$ is the square-wave subcarrier of BOC modulation defined by $sc(t)=sign\left[\sin\left(w_{m}t\right)\right]$ with subcarrier rate of $w_{m}$. Besides,$\alpha_{i}$, $\tau_{i}$, and $\varphi_{i}$ denote the amplitude attenuation, time delay and phase difference for path *i* relative to $s_{0}\left(t\right)$, respectively. Here we can easily get that the time delay $\tau_{0}$ and carrier phase difference $\varphi_{0}$ of the LOS are both zero and amplitude attenuation factor $\alpha_{0}$ is 1. For the sake of brevity and clarity, we just ignore the noise here and will consider its effect in the next section. Then the complex form of the signal after down-conversion can be expressed as:

(2) $$I+jQ=\sum_{i=0}^{P}\alpha_{i}Ac\left(t-\tau_{i}\right)sc\left(t-\tau_{i}\right)e^{j\left(\varphi_{e}+\varphi_{i}\right)}$$

where φ_e_ denotes the difference of carrier phase between the LOS and the locally generated carrier. Generally, without using multipath mitigation methods, the direct signal and multipath signals are all correlated with the local replica in the tracking loop. The final correlation function is written as:

(3) $$R\left(\tau \right)=\sum_{i=0}^{P}\alpha_{i}AR_{0}\left(\tau -\tau_{i}\right)e^{j\left(\varphi_{e}+\varphi_{i}\right)}$$

where $R_{0}\left(\tau \right)$ is the normalized autocorrelation function for BOC signal with multi-peak. According to the Wiener–Khinchin theorem, we obtain the CPSD between incoming signal and local replica as follows:

(4) $$G\left(f\right)=\sum_{i=0}^{P}\alpha_{i}Ae^{j\left(\varphi_{e}+\varphi_{i}\right)}G_{0}\left(f\right)e^{-j2\pi f\tau_{i}}=Ae^{j\varphi_{e}}G_{0}\left(f\right)\left(1+\sum_{i=1}^{P}\alpha_{i}e^{j\varphi_{i}}e^{-j2\pi f\tau_{i}}\right)$$

where is the power spectral density (PSD) function of an ideal BOC signal. It should be noted from (4) that the CPSD is expressed as a product of two parts: The first item is the PSD of an ideal incoming signal with amplitude *A* and carrier phase difference relative to local generated carrier; whereas the second item is exactly the multipath channel transfer function (CTF) given by:

(5) $$\mathrm{CTF}=1+\sum_{i=1}^{P}\alpha_{i}e^{j\varphi_{i}}e^{-j2\pi f\tau_{i}}$$

CTF is the Fourier transform of the time domain channel impulse response (CIR) function [27]. It is a function of $\alpha_{i}$, $\tau_{i}$ and $\varphi_{i}$ which mainly depends on the current multipath channel characteristics and contains all the parameter information of the total *P* multipath signals. So, we can estimate these parameters by carefully analyzing the CTF.

### 2.2. Direct-FFT Method

According to the previous analysis, in order to estimate the multipath parameters using (4), we need to separate CTF from the nominal PSD. A direct way of separating is to perform spectrum division between the CPSD and the nominal PSD of local replica. If we consider the system noise here, (4) can be rewritten as:

(6) $$G\left(f\right)=Ae^{j\varphi_{e}}G_{0}\left(f\right)\left(1+\sum_{i=1}^{P}\alpha_{i}e^{j\varphi_{i}}e^{-j2\pi f\tau_{i}}\right)+G_{noise}\left(f\right)$$

where $G_{noise}\left(f\right)$ is assumed to be the CPSD of the local replica and the additive Gaussian noise (antenna noise and thermal noise from the RF chain) with variance of $\sigma^{2}$. Then the result of spectrum division is shown as:

(7) $$G\left(f\right)/G_{0}\left(f\right)=Ae^{j\varphi_{e}}CTF+G_{noise}\left(f\right)/G_{0}\left(f\right)$$

Generally, there are various methods can be used to extract the multipath parameters from (7). Least square fitting method and its modified method recursive least square (RLS) algorithm [28,29] can be seen as an approach like MEDLL method in the frequency domain by curve fitting in the least squares sense. It is capable to obtain the multipath parameters $\left(\alpha_{i},\tau_{i}\right)$, but unable to get the number of multipath signals. Thus, it is needed to find out ways to estimate or assign the number of multipath signals prior to applying this method. Cepstral analysis and Subspace Estimation Technique, such as the MUSIC method, can be used to search for the multipath number and delay, but they are both insensitive to estimate the amplitude of multipath signals and vulnerable in front of system noise. Besides, they require large-scale matrix transformations and/or singular value decomposition, which will also significantly increase the computational complexity.

Another way to get multipath channel characteristics from CTF is directly translating (7) to the time-domain by taking IFFT of (7). If we assume *A* = 1 and $\varphi_{e}$ = 0, the results after IFFT operation is given by:

(8) $$\begin{array}{l} F^{-1}\left[G\left(f\right)/G_{0}\left(f\right)\right]\\ =\delta \left(t\right)+\sum_{i=1}^{P}\alpha_{i}e^{j\varphi_{i}}\delta \left(t-\tau_{i}\right)+F^{-1}\left(noise\left(f\right)/G_{0}\left(f\right)\right) \end{array}$$

where $F^{-1}$ represents the IFFT operator. We can see from (8) that after IFFT there will be *P* + 1 impulses in the time domain. The first impulse is at the original point with amplitude of 1. It represents the transformation result of LOS. The other *P* impulses correspond to the transformation results of multipath signals. For the *i*-th path, its impulse appears at the epoch where *t* equals to $\tau_{i}$ with amplitude of $\alpha_{i}e^{j\varphi_{i}}$, so we can estimate the parameters of both LOS and reflected signals by searching for these impulses after IFFT. Since LOS is always in front of the multipath signals, it is easy to pick out the impulse of LOS by searching for the first impulse with very large amplitude. Then the time offset of this impulse can be obtained which is exactly the code delay of LOS. After that, we can also search for the other impulses and identify $\tau_{i}$ and $\alpha_{i}$ for each path.

It should be noted that although the method of directly taking IFFT is able to estimate all parameters of multipath signals, it is quite sensitive to the noise. A main reason is that the actual GNSS signal is approximately 20 dB weaker than the thermal noise power in a 2 MHz bandwidth receiver, so the power spectra obtained directly from FFT without statistical averaging will be too noisy to be used for multipath parameter estimation. Besides, to estimate the multipath signals accurately, the system sampling rate must be quite high which results in large data volume. Then, direct taking FFT and IFFT of the entire data will be highly computationally expensive. All the above factors result in its unfeasibility and then prevent it from practical application. 

## 3. Multipath Parameter Estimate Algorithm

### 3.1. Algorithm Description

In order to handle the problems of heavy computational burden and severe noise challenge that the direct-FFT method faces, we develop an averaging-FFT method. It employs the process of statistical averaging to obtain more clear power spectrum of the incoming signal, which contributes to the significant reduction of both computation load and noise effect. This section explains the main principle of the proposed technique and presents the mathematical derivation.

**Figure 1:**
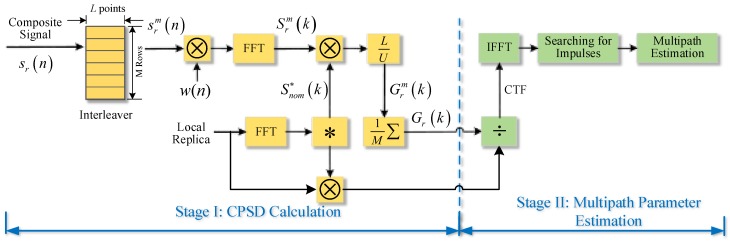
The scheme of the proposed multipath estimation method.

Figure 1 illustrates the scheme of the averaging-FFT method. The proposed method can be summarized into two stages: CPSD calculation and multipath parameter estimation. In the stage I “CPSD calculation”, our goal is to compute the CPSD between incoming signal and local replica. We mainly take measures of segmentation and averaging to minimize the impact of noise. Similar way of processing can also be used for signal quality monitoring [30]. An interleaver is used here to store the consecutive baseband signal in rows and extract each column of rows for parallel computing. In practice, interleaver is implemented by mass storage devices like SDRAM in which *M* different rows store *M* consecutive segments, and *L* columns of the rows are sequentially read out for windowing and FFT [31,32]. Thus, this phase can be stated as follows:

- Step 1: Let the baseband signal after sampled and down-conversion first pass an interleaver to be divided into *M* segments with *L*-point length for each segment;
- Step 2: Add the time-domain hamming window to each segment to reduce the effect of leakage and truncation;
- Step 3: Perform FFT to obtain the frequency spectrum of each segment;
- Step 4: Multiply the frequency spectrum by the conjugate spectrum of local replica to compute the CPSD of each segment;
- Step 5: Average the total M segments to get the final CPSD.

The second stage “multipath parameter estimation” can be divided into 3 steps. First, the CPSD obtained from state I is divided by the nominal PSD of local replica in the frequency domain to get the CTF. Then, we take the IFFT of CTF to obtain the channel impulse response function in the time domain. Finally, we search for the time offset and amplitude of each impulse and output the estimated multipath parameters. Next, the thorough mathematical derivation for both two stages is given in the following part.

### 3.2. CPSD Calculation

For a sample set of the input baseband signal $s_{r}\left(0\right),\ldots ,s_{r}\left(N-1\right)$ with total length of *N* points, it can be expressed as:

(9) $$s_{r}\left(n\right)=s\left(n\right)+noise\left(n\right)$$

where s(n) is the clear signal given by (2) and noise(n) denotes the thermal noise following normal distribution: $noise\left(n\right)\sim N\left(0,\sigma^{2}\right)$.

**Figure 2:**
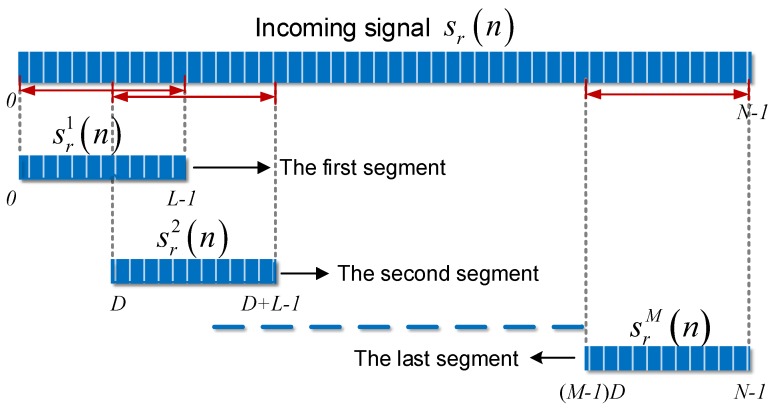
Explanation of segmentation for the proposed method. The entire signal falls into M segments.

The segmentation and overlap are illustrated in Figure 2. The total signal is first divided into *M* segments. The starting points of these segments are D units apart. Then we will have (M−1)D+L=N and the overlap rate *r* is (1−D/L)×100%. If we let $s_{r}^{1}\left(n\right)$ denote the first segment with *n* = 0, 1, … *L* − 1, namely $s_{r}^{1}\left(n\right)=s_{r}\left(n\right)$, *n* = 0, 1,… *L* − 1. Similarly, we obtain $s_{r}^{2}\left(n\right)=s_{r}\left(n+D\right)$, and at last $s_{r}^{M}\left(n\right)=s_{r}\left(n+(M-1)D\right)$.

After segmentation, the samples of each segment will be weighted by a window function w(n) for the sake of reducing the harmful effects of spectral leakage. Let $S_{r}\left(k\right)$, S(k) and Noise(k) be the discrete Fourier transform coefficients of $s_{r}\left(n\right)$, clear signal and system noise at frequency bin k, respectively. The size of this frequency bin is defined by $f_{s}/L$, where $f_{s}$ is the system sampling rate. For the *m*-th segment, the discrete Fourier transform coefficients can be calculated by:

(10) $$S_{r}^{m}\left(k\right)=S^{m}\left(k\right)+Noise^{m}\left(k\right)$$

where:

(11) $$S^{m}\left(k\right)=\frac{1}{L}\sum_{n=0}^{L-1}s^{m}\left(n\right)w\left(n\right)e^{-j\frac{2\pi }{L}kn}$$

(12) $$Noise^{m}\left(k\right)=\frac{1}{L}\sum_{n=0}^{L-1}noise^{m}\left(n\right)w\left(n\right)e^{-j\frac{2\pi }{L}kn}$$

and $j={\left(-1\right)}^{1/2}$. For the *m*-th segment $s_{r}^{m}\left(n\right)$, the CPSD is given by:

(13) $$G_{r}^{m}\left(k\right)=\frac{L}{U}S_{r}^{m}\left(k\right)S_{nom}^{*}\left(k\right)$$

where ∗ represents conjugate operation and $S_{nom}\left(k\right)$ is the discrete Fourier transform coefficient of ideal BOC signal with *L* points. In order to reduce the effect of window on the total power, the CPSD is further divided by a coefficient *U*, which is given by:

(14) $$U=\frac{1}{L}\sum_{n=0}^{L-1}w^{2}\left(n\right)$$

The final CPSD estimate $G_{r}\left(k\right)$ can be calculated by averaging $G_{r}^{m}\left(k\right)$, *m* = 1, 2, …, *M*:

(15) $$G_{r}\left(k\right)=\frac{1}{M}\sum_{m=0}^{M-1}G_{r}^{m}\left(k\right)$$

The CPSD obtained from (15) is less noisy. It effectively enhances the robustness of the multipath parameter estimation in the next subsection. It should be noted that a nonrectangular time window, such as hamming window, is used to reduce spectral leakage by attenuating both ends of each segment. This unequal weighting of the samples increases the uncertainty of the power spectrum estimation considerably. So we employ a certain level of overlap between two adjacent segments to eliminate this unequal weighting, which is helpful to reduce the estimation variance. A overlap rate of 50% could be a reasonable value.

### 3.3. Multipath Parameter Estimation

Employing the CPSD obtained during stage I, we perform multipath parameter estimation in stage II. When we consider (10) and (4), and also assume *A* = 1 and $\varphi_{e}$ = 0, the CPSD of the *m*-th segment can also be written as:

(16) $$\begin{array}{ll} G_{r}^{m}\left(k\right) & =\frac{L}{U}\left(S^{m}\left(k\right)S_{nom}^{*}\left(k\right)+Noise^{m}\left(k\right)S_{nom}^{*}\left(k\right)\right)\\ & =G^{m}\left(k\right)+G_{noise}^{m}\left(k\right) \end{array}$$

where:

(17) $$G^{m}\left(k\right)=G_{0,L}\left(k\right)\left(1+\sum_{i=1}^{P}\alpha_{i}e^{j\varphi_{i}}e^{-j2\pi \frac{k}{L}f_{s}\tau_{i}}\right)$$

and $G_{0,L}\left(k\right)$ is the PSD of an ideal BOC signal with *L*-point length. Finally, the averaged CPSD using total *M* segments is given by:

(18) $$\begin{array}{ll} G_{r}\left(k\right) & =\frac{1}{M}\sum_{i=0}^{M-1}G_{r}^{m}\left(k\right)\\ & =G_{0,L}\left(k\right)\left(1+\sum_{i=1}^{P}\alpha_{i}e^{j\varphi_{i}}e^{-j2\pi \frac{k}{L}f_{s}\tau_{i}}\right)+\frac{1}{M}\sum_{i=1}^{M}G_{noise}^{m}\left(k\right) \end{array}$$

In order to remove the effect of the PRN code, averaged CPSD is divided by the nominal PSD. Then we obtain the CTF as well as a noise term, which is given by:

(19) $$\frac{G_{r}\left(k\right)}{G_{0,L}\left(k\right)}=\left(1+\sum_{i=1}^{P}\alpha_{i}e^{j\varphi_{i}}e^{-j2\pi \frac{k}{L}f_{s}\tau_{i}}\right)+\frac{1}{M}\sum_{i=1}^{M}\frac{G_{noise}^{m}\left(k\right)}{G_{0,L}\left(k\right)}$$

Finally, we take the IFFT operation of the result and obtain the impulse response function in time domain as follows:

(20) $$F^{-1}\left[\frac{G_{r}\left(k\right)}{G_{0,L}\left(k\right)}\right]=\delta \left(t\right)+\sum_{i=1}^{P}\alpha_{i}e^{j\varphi_{i}}\delta \left(t-\tau_{i}\right)+F^{-1}\left[\frac{1}{M}\sum_{i=1}^{M}\frac{G_{noise}^{m}\left(k\right)}{G_{0,L}\left(k\right)}\right]$$

Consider the result in (20). The useful parts, the first two parts, are independent of *M*, whereas the noise term is the averaging of *M* terms. Since $G_{0,L}\left(k\right)$ and $G_{noise}^{m}\left(k\right)$ are both computed for *L* points, the result $G_{noise}^{m}\left(k\right)/G_{0,L}\left(k\right)$ is equivalent to $noise\left(f\right)/G_{0}\left(f\right)$ in (8). Further, $G_{noise}^{m}\left(k\right)$ is given by $Noise^{m}\left(k\right)S_{nom}^{*}\left(k\right)$ and $G_{0,L}\left(k\right)$ is defined as $S_{nom}\left(k\right)S_{nom}^{*}\left(k\right)$. Then the *m*-th noise term $G_{noise}^{m}\left(k\right)/G_{0,L}\left(k\right)$ can be simplified as $Noise^{m}\left(k\right)/S_{nom}\left(k\right)$, where $Noise^{m}\left(k\right)$ is varying in amplitude and phase for different *m*, so we can see that the noise terms are isotropic, and thus the averaging operation $\frac{1}{M}\sum_{i=1}^{M}\left(\cdot \right)$ finally reduces the noise, making sure the noise power after averaging is smaller than $G_{noise}^{m}\left(k\right)/G_{0,L}\left(k\right)$ and also the results in (8), which brings the performance gain compared with the direct-FFT method.

In order to declare the presence of multipath, we need to set a threshold. Once the amplitude of a certain impulse excesses the threshold, a multipath-presence decision is made and a ray of multipath is found; otherwise, a multipath-absence decision is made. Generally, the threshold value is a function of the designated false alarm probability and the level of thermal noise. For a designated C/N_0_, the final false alarm rate (*P_fa_*) and detection rate (*P_d_*) can also be seen as functions of the threshold value. In order to calculate *P_fa_* and *P_d_*, the distributions of the amplitude of impulses for both multipath-absent and multipath-present circumstances have to be obtained. However, the distributions of the IFFT results are quite complicated. Besides, they also depend on the window size used in the method, the receiver bandwidth, sampling frequency and the specific multipath circumstance. It is impractical to derive the analytical expression of the probability density functions.

One possible way to set the threshold is using the statistical method. The distribution of the IFFT result is first analyzed and computed under the circumstance of clear signal without multipath. The impulse of LOS is removed before calculation. Thus, we can obtain the distribution of the IFFT result that only depends on the system noise. Then for a given false alarm probability, the corresponding threshold is also determined. One of the benefits of such process is we can set the threshold without the knowledge of the exact mathematical expression of the probability distribution. 

In this paper, as the main purpose is not at multipath detection but parameter estimation, we employ a simplified way to determine the presence of multipath as well as LOS. When to estimate the code delay of LOS in the multipath environment, we just need to search for the impulse with the largest amplitude basing on the fact that reflected signals must be weaker than the LOS signal. However, when to estimate the parameters of all multipath signals, we need to set the threshold with a pre-set false alarm probability just as described in the previous paragraph to find all impulses that excess the threshold. Then, the first impulse corresponds to the LOS and the other impulses represent the reflected signals. Thus in this way, we can estimate the parameters of all paths without need of a-priori assumption about the number of extra paths like MEDLL method. 

### 3.4. Computation Complexity Analysis

As mentioned in the Introduction section, computation complexity is one of main factors that limit the practical application of parametric multipath mitigation techniques. For subspace estimation techniques, either MUSIC or ESPRIT, all need singular value decomposition or eigenvalue decomposition computation, which is the key step when implemented by Field Programmable Gate Array (FPGA). Singular value decomposition includes a large amount of nonlinear computation with time complexity of $O\left(n^{3}\right)$. However, the baseband signal is generally sampled by a relatively high sampling rate (such as 20 MHz is used in this paper). If the entire signal length is 10 ms, the total number of sample points *N* can be as large as 2 × 10^5^. Thus, it is almost impossible for hardware to process such a signal in real time. The problem of high computation complexity also plagues the maximum likelihood approach, such as MEDLL method. MEDLL estimates the multipath parameters by iteration operations. As the number of extra paths goes up, the total computational cost will increase exponentially.

Motivated by the search of a simpler parametric technique for BOC signals, the frequency-domain approach we proposed in this paper does not require any eigenvalue decomposition or iteration operations. The main complexities are FFT operations and one-time IFFT operation which are easy for FPGA to fulfil. Besides, in comparison with the direct-FFT method, the proposed averaging-FFT method also excels at saving computational resources. The computation load it takes for the two stages is calculated below.

Typically, for a hardware, *N*-point FFT operation requires $0.5*N\log_{2}N$ times multiplication together with $N\log_{2}N$ times addition. By recalling Figure 2, the total segment number *M* can be expressed as (N−rL)/((1−r)L). For *M* times *L*-point FFT operation, the total computation load includes $C_{m1}^{I}$ times multiplication and $C_{a1}^{I}$ times addition, which is given by:

(21) $$\begin{array}{l} C_{m1}^{I}=M\cdot \frac{L}{2}\cdot \log_{2}L=\frac{N-rL}{2\left(1-r\right)}\log_{2}L\\ C_{a1}^{I}=M\cdot L\cdot \log_{2}L=\frac{N-rL}{\left(1-r\right)}\log_{2}L \end{array}$$

Besides, extra computation is required to add windows and perform averaging. This part of computation amount contains $C_{m2}^{I}$ times multiplication and $C_{a2}^{I}$ times addition given by:

(22) $$\begin{array}{l} C_{m2}^{I}=M\cdot L=\frac{N-rL}{\left(1-r\right)}\\ C_{a2}^{I}=L\;\left(M-1\right) \end{array}$$

So for state I, the total required multiplications $C_{m}^{I}$ is $C_{m1}^{I}+C_{m2}^{I}$, and addition $C_{a}^{I}$ is $C_{a1}^{I}+C_{a2}^{I}$.

For stage II, total operations include one times *L*-point division and one times *L*-point IFFT operation with calculated amount of $C_{m}^{II}$ times multiplication and $C_{a}^{II}$ times addition given by:

(23) $$\begin{array}{l} C_{a}^{II}=L+\frac{L}{2}\cdot \log_{2}L\\ C_{m}^{II}=L\cdot \log_{2}L \end{array}$$

Finally, the overall computation amount is the sum of the above two parts, namely we totally need $C_{m}=C_{m}^{I}+C_{m}^{II}$ times multiplication and $C_{a}=C_{a}^{I}+C_{a}^{II}$ times addition. After simplifying the equations, we have $C_{m}$ and $C_{a}$ as:

(24) $$\begin{array}{l} C_{m}=\frac{N+L-2rL}{2\left(1-r\right)}\log_{2}L+\frac{N+L-2rL}{\left(1-r\right)}\\ C_{a}=ML\log_{2}L=\frac{N+L-2rL}{\left(1-r\right)}\log_{2}L+\frac{N+L-2rL}{\left(1-r\right)} \end{array}$$

whereas for the direct-FFT method, it totally takes one times *N*-point FFT, division and IFFT, so the total computation amount is $N\cdot \log_{2}N+N$ times multiplication together with $2N\cdot \log_{2}N$ times addition.

In order to show the computation load reduction clearly, we assume the entire data length is 10 ms, sampling rate is 10 MHz and *L* is 128. The overlap rate *r* is set to 50%. Under these conditions, we compute the ratio of computation amount of two methods. For multiplication, the ratio is $C_{m}/\left(N\log_{2}N+N\right)\times 100\%\approx 51.1\%$, and for addition, the ratio is $C_{a}/\left(2N\log_{2}N\right)\times 100\%\approx 48.2\%$. We can see that for this case, the proposed method just needs approximately half of the overall computation amount. 

### 3.5. Effect of Window Length L

The window length *L* is of crucial importance to the proposed method. It not only affects the computation amount as we mentioned above, but also has direct effect on other two aspects: the robustness to the noise and maximum detectable multipath delay of the proposed method. By analyzing the effect of *L*, we can have a better understanding of the new technique and apply it properly under various noise levels.

As mentioned above, the number of segments *M* is a function of *L*. For a given overlap rate *r*, a smaller *L* means a larger *M*. Then the noise will be smoothed for more times and the robustness of the method is thus improved accordingly. 

It should be noted that the robustness improvement comes along with the decrease of maximum detectable multipath delay. The maximum detectable multipath delay $\tau_{\max}$ is defined as the maximum range of the multipath delay that the method is able to detect. It can be expressed by:

(25) $$\tau_{\max}=L/f_{s}T_{c}$$

So for a multipath component with a code delay of $\tau_{\Delta }$ in units of code chips, we need to make sure $\tau_{\Delta }<\tau_{\max}$ to keep it detectable. Generally, this requirement is easy to satisfy. The range of multipath delay is typically varying from 0 to 2 chips, because if the path delay is too large (such as larger than 2 chips), this ray will suffer from severe amplitude attenuation and thus have little impact on the positioning accuracy of user receivers. Considering the worst circumstance that $\tau_{\Delta }$ is 2 chips and the sampling rate $f_{s}$ is 20 MHz, as long as the *L* is larger than 40 points, $\tau_{\max}$ will exceed 2 chips and this reflected ray can be detected.

## 4. Performance Evaluation of Parameter Estimation

In the previous section, we explained the principle of the proposed method for multipath parameter estimation using processes of segmentation and averaging. This section gives the simulation results in comparison with the direct-FFT method.

### 4.1. Comparison between Direct-FFT Method and the Proposed Method

Although the direct-FFT method has a good spectrum resolution, it suffers from significant estimation variance. Besides, as it employs a rectangular time-window to restrict the infinite time signal to a finite time horizon, it faces spectrum leakage which further enhances the amplitude of the useless side peaks of the spectrum. Whereas the proposed technique uses a nonrectangular time window to achieve good property for leakage reduction. The time averaging also minimizes the effect of noise.

**Figure 3:**
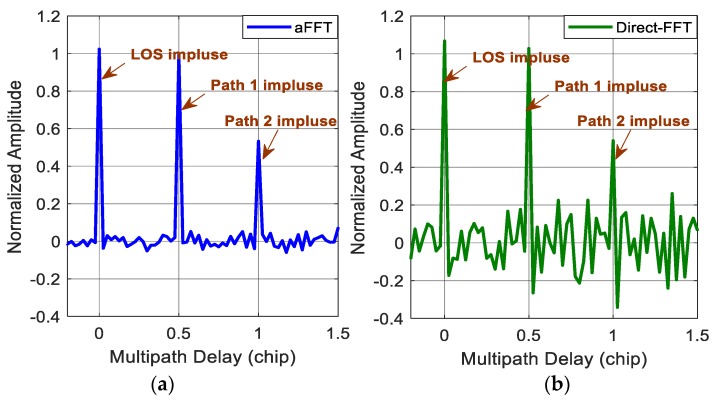
(**a**) Impulse responses of the averaging-FFT method with C/N_0_ of 45 dB-Hz; (**b**) impulse responses of the direct-FFT method with C/N_0_ of 45 dB-Hz.

**Figure 4:**
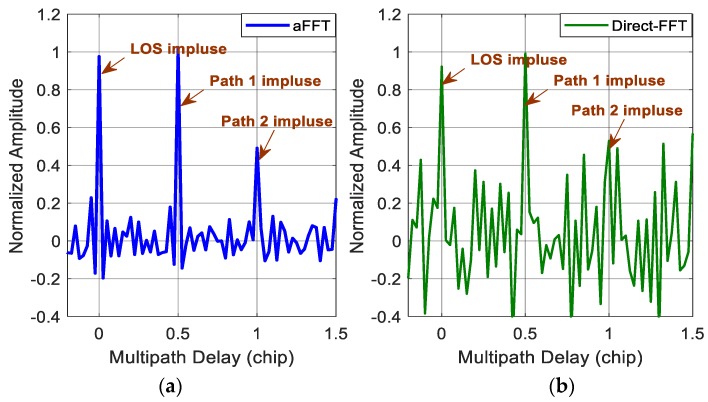
(**a**) Impulse responses of the averaging-FFT method with C/N_0_ of 38 dB-Hz; (**b**) impulse responses of the direct-FFT method with C/N_0_ of 38 dB-Hz.

Figure 3 and Figure 4 show the impulse responses of the direct-FFT method and the averaging-FFT method with a window time L of 256 points for C/N_0_ of 45 and 38 dB-Hz, respectively. In the simulations, we assume two multipath signals. The first one has the same power as the direct signal with a time delay of a half chip and the second one has attenuation factor α of 0.5 and time delay τ of one chip. Because of the noise, the normalized amplitude of three impulses (one is for LOS and the other two are for multipath signals) after IFFT are random variables fluctuating at the value of 1 and 0.5, instead of constants. When C/N_0_ is 45 dB-Hz, the two methods are all able to estimate the amplitude and time delay of both the direct signal and two multipath signals well. But, the noise variance of the direct-FFT method is obviously higher than the aFFT method. Whereas for the case of 38 dB-Hz, the two methods both get worse. The aFFT method is still able to provide the information of multipath parameters accurately. But the direct-FFT method can hardly distinguish the impulses from noise, especially for the second reflected signal with smaller amplitude of 0.5. So for this method, longer data are needed to achieve the performance improvement and minimize the noise. But meanwhile, the required computational load will increase accordingly, and the real-time capability will definitely get worse. Consequently, the implement of direct-FFT method requires relatively good C/N_0_ condition, whereas the proposed method can keep acceptable performance in poor noise conditions.

### 4.2. Parameter Estimation Results

By searching for the impulses after IFFT, we can easily obtain the time delay $\tau_{i}$ of both LOS and the reflected signals. As the reflected signals are always later than the LOS signal to reach the receiver antenna, so it is easy to distinguish LOS from multipath signals. Also, when to estimate the parameters of multipath signals, we just ignore the first impulse and get the impulses corresponding to multipath signals.

In this subsection, we tested the performance of estimating multipath delay using the averaging-FFT method, direct-FFT method and MEDLL on BOC(1,1) signal. The setting of simulation parameters is concluded in Table 1. In this test, for convenience, we just retain one multipath signal with an attenuation of −3 dB. The according time delay $\tau_{1}$ is set to vary from 0.1 to 1 code chip. The window length *L* of the aFFT method is set to 128 points and the total signal length is 40 ms. C/N_0_ is set to 36 dB-Hz, which is a relative low value for a GNSS receiver on the ground. For each value of $\tau_{1}$, multipath estimation has been performed for 1000 times, and the estimation results are used to calculate the estimation deviation.

**Figure 5:**
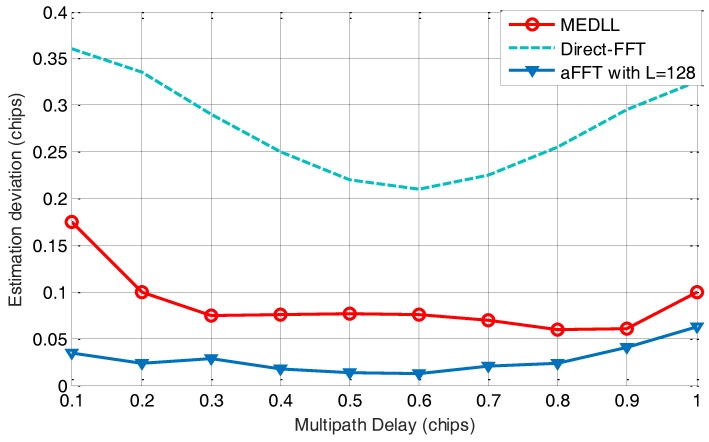
Estimation deviation of estimated code delay with τ varying from 0.1 to 1 chip.

Figure 5 shows the estimation deviation of the multipath delay using three methods. We can see that the averaging-FFT method keeps the smallest estimation deviation compared with the other two methods. The maximum value is slightly more than 0.05 code chip. MEDLL method also has an acceptable estimation deviation curve. But when $\tau_{1}$ is smaller than 0.3, its estimation error gets larger, which indicates that MEDLL may not perform well when the reflected signal is too close to the LOS. Finally, the direct-FFT method shows the worst estimation performance, due to its high vulnerability to the noise.

### 4.3. Tests with Real Data

In the previous section we showed the theoretical parameter estimation capability of the proposed method. To make the results more persuasive, we verified it with real GPS signals. The dataset used here is the so-called Texas Spoofing Test Battery (TEXBAT) [33]. It is a test battery of real cases publicly provided by the University of Texas at Austin. It is originally collected to demonstrate the vulnerability of GNSS in face of a spoofing attack and test the capability of spoofing detection techniques. However, numerous papers have mentioned that the structure of the spoofing signals is quite similar with multipath signals [34,35]. Even the same mathematic model is employed to characterize multipath and spoofing signals. This allows us to use the TEXBAT dataset as a set of multipath signal records for real data tests. Besides, the dataset only contains one secondary path signal and its behavior is known to us, which makes it quite appropriate for the verification of the performance of the proposed aFFT method. Signals were tracked using a GPS software receiver. The setting of parameters is concluded in Table 2. The signal modulation type used here is BPSK modulation (GPS C/A signal). The data was collected by a sampling rate of 25 MHz. The total signal length to perform one time multipath estimation is set to 20 ms, and the window size *L* is 128 points.

**Figure 6:**
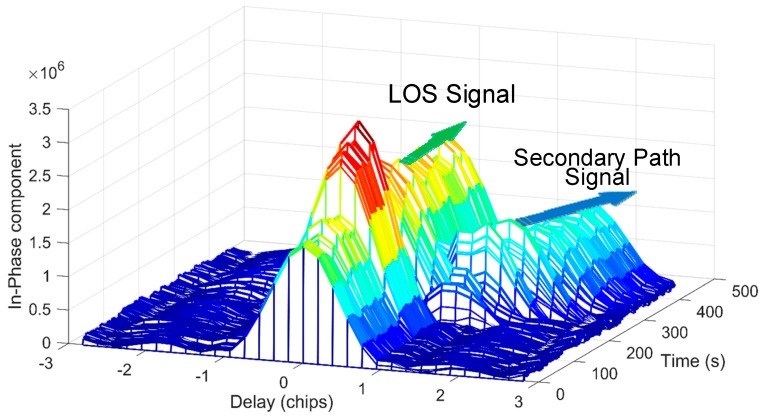
In-Phase correlation function of TEXBAT dataset.

The scenario we used is number 6 of a total of six different cases. In this scenario, a signal with the same structure as the real GPS signal is broadcast to reach the antenna of the victim receiver. The mixture of the LOS signal and the counterfeit signal can be seen as multipath signals. The behavior of the mixture signal in I channel is illustrated in Figure 6. This scenario includes a dynamic secondary path signal with a matched power advantage over the LOS signal (+0.8 dB). Compared with static scenarios, the receiver used to collect this GPS data is moving or dynamic. Thus, it can be used to verify the performance of the proposed method in dynamic conditions. In order to reduce the effect, the total signal length for one time process should not be too long, and the renewal cycle and noise bandwidth of the code and carrier loop should be a little larger.

**Figure 7:**
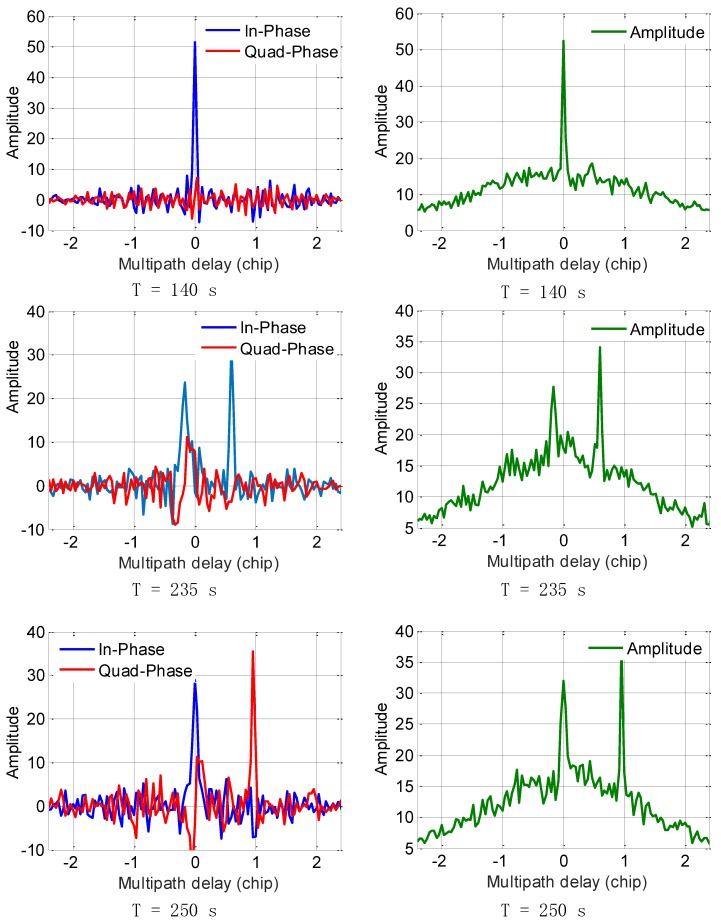
Impulse responses of the proposed averaging-FFT method over TEXBAT dataset.

The LOS signal and multipath signal starts separating at the 150th second. In order to show the parameter estimation ability of the proposed aFFT method, we provide the impulses at the 140th, 235th, and 250th seconds, respectively as shown in Figure 7. The three subfigures on the left side show the in-phase and quadrature phase impulses after IFFT operation, and the subfigures on the right side illustrate the amplitude (absolute value) of the IQ composite impulses. Employing both the impulses of I/Q channel, we can easily obtain the current carrier phase difference between LOS signal and multipath signals. From Figure 7, we can see clearly the total changing process, which demonstrates the effectiveness of the proposed method. The impulses of both LOS signal and the extra ray can be easily detected by a threshold, and we can characterize and distinguish both LOS signal and multipath signals using the averaging-FFT method. Besides, the carrier phase difference between LOS signal and multipath signal has much effect on the I/Q channel energy distribution. We can see from the results of T = 250 s that the multipath signal is totally orthogonal to the LOS signal, so the vast majority of the multipath signal energy is distributed in the Q channel, but this phase difference will not affect the parameter estimation of multipath delay when we use the absolute value of I/Q channel impulses as shown in the subfigures on the right side.

However, compared with the theoretical results provided in Figure 3 and Figure 4, the impulses seem noisier. This probably due to the non-ideal characteristic of real data and the specific receiver setting. Besides, the carrier phase difference between LOS signal and multipath signal would not be a constant value compared with the ideal condition. It is changing continuously. All the above factors lead to a slight performance loss. And all multipath estimation techniques, not just the aFFT method, have to face these challenges.

Nevertheless, the results still show the effectiveness and feasibility of the proposed method on real data even in the dynamic environments. We can easily estimate the code delay using the impulses processed by the averaging-FFT method. Besides, if we enlarge the signal length for each estimation (here is 20 ms) or choose a shorter window size (here is 128 points), as analyzed in the paper, the total performance can be improved further.

## 5. Multipath Mitigation Results

According to the analysis above, the implementation of segmentation and time averaging helps the frequency domain multipath estimation method achieve better performance. Even in poor C/N_0_ conditions, we can further improve the estimation accuracy by reducing the length of *L*. Since multipath parameter estimation serves the goal of multipath mitigation on BOC signals, it is meaningful to evaluate the final ranging accuracy improvement in the multipath conditions using the proposed technique. So in this section, we plotted the root-mean-square error (RMSE) curves of code tracking and presented the comparison results of different multipath mitigation techniques. However, as our target signals are BOC-modulated signals, even high-order BOC signals, the TEXBAT dataset of GPS C/A signals would not be appropriate. So, the performance evaluation in this section was implemented by simulations. This scheme for performance evaluation had also be adopted in papers, such as [16,36]. One of the benefits of such process is any other error sources can be excluded except multipath error.

**Figure 8:**
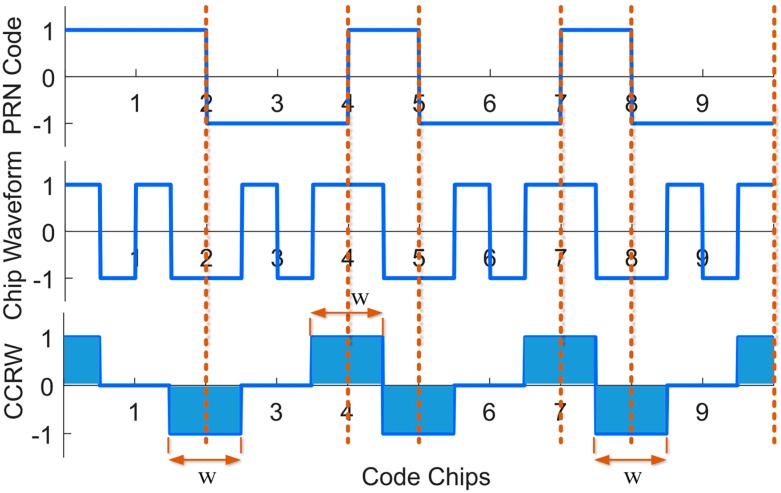
Illustration of CCRW method used in the following simulations.

**Figure 9:**
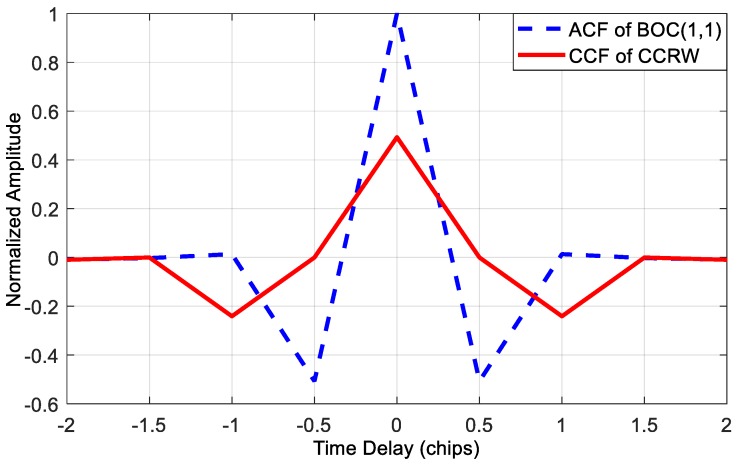
The CCF between BOC(1,1) signal and local CCRW signal.

In this part, we mainly consider three different methods which can be used for multipath mitigation for BOC signals, i.e., the proposed method, MEDLL, and CCRW. They are classified into two groups. The first two techniques are parametric. Particularly, the MEDLL is among the most popular approaches of this type. CCRW is non-parametric and the best representative of this type. As mentioned in the Introduction section, there are various local reference waveforms among the CCRW family. Without loss of generality, the CCRW method here is implemented by reference to [7,37]. Figure 8 and Figure 9 show the local waveform signal and the cross correlation function between the reference waveform and the nominal BOC(1,1) signal, respectively. Non-zero values of the local CCRW signal appear only at the PN code transitions. The width of these gated waveform *w* is set to n/m PN code chips for a BOC(*m*,*n*) signal. So for BOC(1,1), *w* is one PN code chip.

Besides, it is should be noted that the two parametric techniques, MEDLL and aFFT method, achieve the goal of multipath mitigation by means of estimating the multipath parameters. We can obtain the code phase observations by directly estimating the time delay $\tau_{0}$ of LOS. This is equivalent to the output of prompt correlator in a conventional tracking loop. The estimate of $\tau_{0}$ will be further used to calculate the range estimates. As mentioned above, RMSE was used in this paper to indicate the multipath estimator performance. In order to calculate the RMSE, the trial of multipath estimation has been performed for a large number of times, such as 1000 times here. The mathematical expression of RMSE is generally defined as follows:

(26) $$\mathrm{RMSE}=\sqrt{\frac{\sum_{p=1}^{P}{\left({\hat{r}}_{p}-r_{0}\right)}^{2}}{P}}$$

where *P* denotes the total times of trails, ${\hat{r}}_{p}$ represents the range estimate of the p-th trail. $r_{0}$ is the actual rang value defined directly as $r_{0}=\tau_{0}\cdot c$, where $\tau_{0}$ is the pre-set delay of LOS and *c* is the speed of light. In the simulations of section “Multipath Mitigation Results”, $\tau_{0}$ was set to 0 for convenience. But if we try to estimate the delay of a multipath signal, it should be set to the actual code delay of that path.

At the same time, in order to show the universality and practicality of the proposed method, we set up three different simulation scenarios in this section: (1) single-path channel; (2) three-path channel; and (3) high-order BOC signals. They are all static scenarios.

Most of the multipath mitigation algorithms in the previous literatures are tested in the presence of a few secondary paths (three or less). Whereas in real urban or indoor environments, the multipath condition can be much harsher. The multipath severity is reflected in two aspects: (1) the number of paths may be far more than three; and (2) reflected signals are very powerful relative to the LOS with a quite small time delay. Generally, the performance of conventional methods may deteriorate dramatically in these severe multipath environments. So we particularly set up the simulation scenario 2 with a three-path channel to test the proposed method in the presence of severe multipath.

Besides, BOC modulation type is also considered as an important factor affecting the multipath mitigation performance of different methods. Existing multipath mitigation methods may not be optimal for different BOC modulations, especially for some high-order BOC signals. Therefore in the last scenario, we also evaluated the multipath mitigation capability on BOC(14,2) and BOC(15,2.5) modulations.

### 5.1. Scenario 1: Single-Path Channel

In this subsection, we tested the code tracking performance of the above three methods in the presence of a single multipath component. This test can be seen as a basic controlled trial whose results can be used to make a comparison with that obtained from the next two tests. The setting of simulation parameters is given in Table 3. A multipath component with attenuation of −3 dB and delay of 75 m is added. The receiver intermediate frequency sampling rate is set to 20 MHz, which means the double-sided bandwidth is 20 MHz. Particularly, we choose two window sizes, *L* = 64 points and *L* = 128 points, to make a comparison with other methods, which helps us get a better understanding of the effect of window size *L*. The total signal length herein is 40 ms and overlap rate is 50%. Generally, long-term data can be processed to achieve performance gain. However, for conventional anti-multipath techniques in correlation domain, such as CCRW technique, navigation bit transition is an issue that cannot be ignored and needs special treatment. But the IFFT-based method does not need to consider the effect of navigation bit transition, so long-term data as long as several seconds would be employed for multipath, which is also an advantage of the frequency processing technique over the conventional correlation domain anti-multipath techniques.

**Figure 10:**
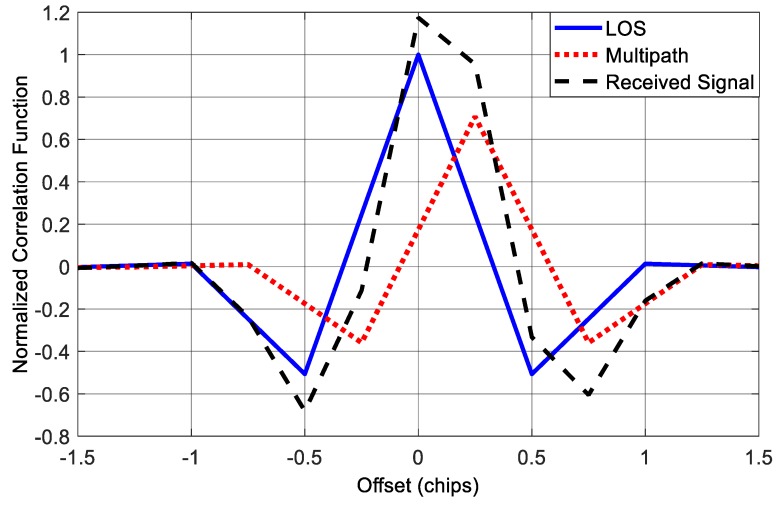
Correlation functions in the presence of single multipath component.

Figure 10 shows the correlation function corresponds to the LOS component for this case. We can see that the correlation peak of the received signal is no more symmetry, which will bring errors to the conventional tracking loops, as well as the multipath mitigation methods based on measuring the correlation function.

**Figure 11:**
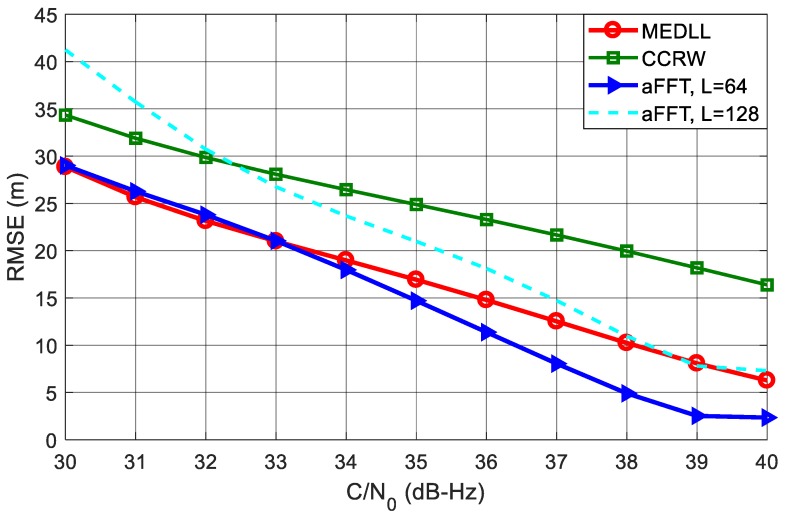
RMSE curves of averaging-FFT, MEDLL and CCRW methods with C/N_0_ varying from 30 dB-Hz to 40 dB-Hz.

Figure 11 shows the RMSE curves of code delay estimation using different algorithms. The C/N_0_ is varying from 30 dB-Hz to 40 dB-Hz. It can be seen that the aFFT method with *L* = 64 outperforms all other algorithms. Particularly when C/N_0_ is 40 dB-Hz, the RMSE is just approximate 2.5 m; whereas for MEDLL and CCRW, the RMSE is around 6 m and 16 m. CCRW method, by contrast, has the worst performance. This is probably because CCRW method employs the correlation shape to estimate code delay. Since the correlation peak has been distorted in the presence of the multipath signal, the CCRW suffers from a significant bias even if without noise. Besides, we can also see that compared with *L* = 64, the RMSE of aFFT with *L* = 128 has a degradation of about 5 m to 8 m. This confirms the analysis in subsection entitled *Analysis of window length L* that smaller *L* helps reduce the noise effect.

### 5.2. Scenario 2: Three-Path Channel

**Figure 12:**
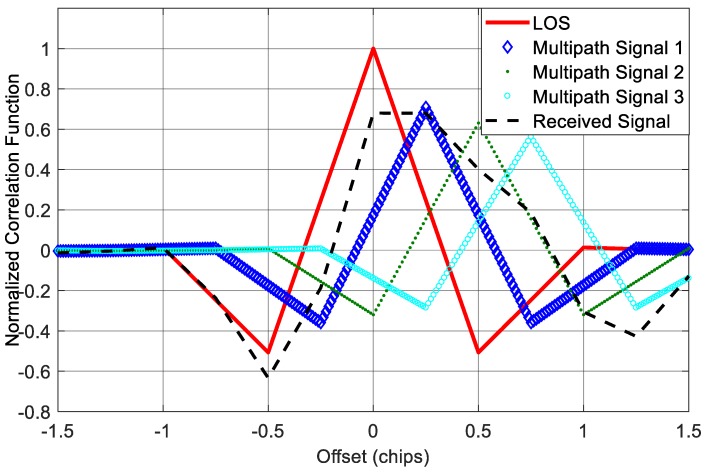
Correlation functions in the presence of three multipath components.

In the simulations above, we consider a single-path situation. However, this is not the case for some applications, especially for applications in urban and indoor environments where the number of paths could be much larger. So in the following test, we added extra two paths to the received signal in *Scenario 1*, and thus we had a LOS contaminated by three multipath signals. The first multipath component is identical to the multipath signal in *Scenario 1*; the second and third multipath components have 4 dB and 5 dB attenuation with 150 m and 225 m delay relative to the LOS component, respectively. The delays are approximately equivalent to half and 0.75 code chip for BOC(1,1) signal. Figure 12 shows the correlation function in this situation. We can see that when there are three multipath components, the final correlation function of the incoming signal is not only seriously distorted, but also shift to the right, which will result in significant ranging bias to the receiver.

**Figure 13:**
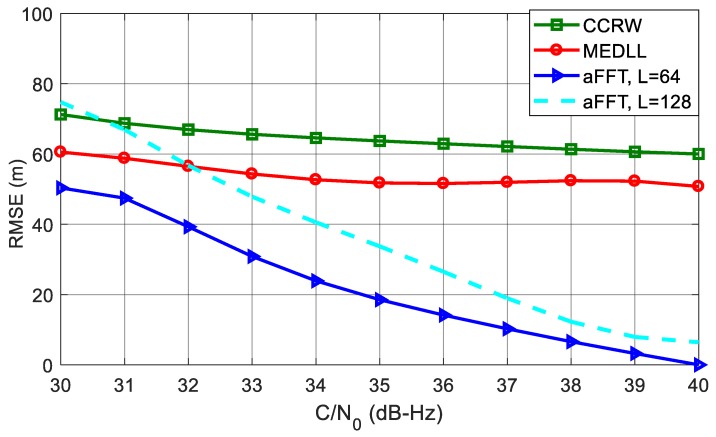
RMSE curves of averaging-FFT, MEDLL and CCRW methods in the presence of three paths.

Figure 13 plots the RMSE cures of different methods in the presence of three multipath signals. Compared with the single-path test, all four RMSE curves of code phase estimation increase dramatically. CCRW and MEDLL both keep very large RMSE values, and this condition has not been obviously improved with the increase of C/N_0_. Whereas for the two aFFT methods, the RMSE curves go down significantly as C/N_0_ gets larger. When the C/N_0_ comes to 40 dB-Hz, the RMSE curves of two aFFT method are both lower than 10 meters. For *L* = 64, the RMSE is almost 50 m smaller than MEDLL and 60 m smaller than CCRW, so the aFFT method is robust enough to the number of the multipath signals, but for MEDLL and CCRW, once there are many secondary rays in the environment, the performance of code delay estimation will deteriorate dramatically.

### 5.3. Scenario 3: High-Order BOC Signals

Because of the excellent ranging accuracy and good spectral separability from the existing GNSS signals, some high-order BOC signals are adopted for military or safety critical applications. For example, the Chinese Beidou system employs the BOC(14,2) modulation of B1 frequency point as a new military signal and the Galileo system L1 PRS is a BOC(15,2.5) signal. However, these high-order BOC signals also bring a challenge that their auto-correlation functions have more secondary peaks compared with low-order BOC signals, such as BOC(1,1) signal, which makes it more difficult to track the LOS in a multipath environment. In this test, we mainly considered two high-order BOC signals, BOC(14,2) and BOC(15,2.5), to show the applicability of different multipath mitigation techniques on high-order BOC signals. Besides, in order to facilitate comparison and analysis, the setting of multipath signal keeps consistent with the single-path channel scenario, except that the system sampling rate is set to 40 MHz due to the wider frequency spectrum of high-order BOC signals.

**Figure 14:**
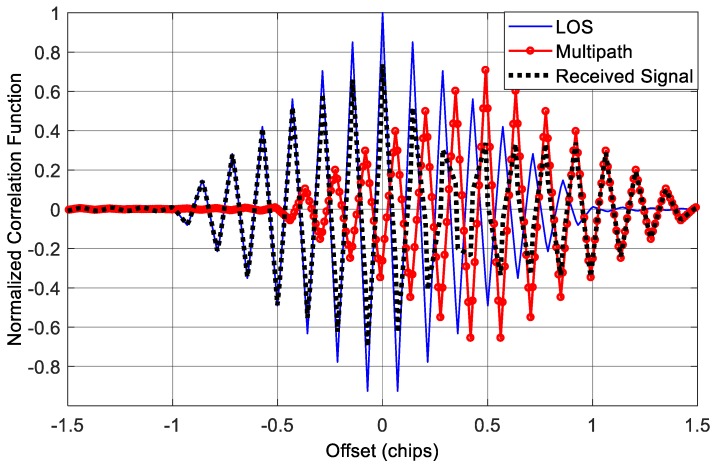
Correlation function of BOC(14,2) signal in the presence of single multipath component.

**Figure 15:**
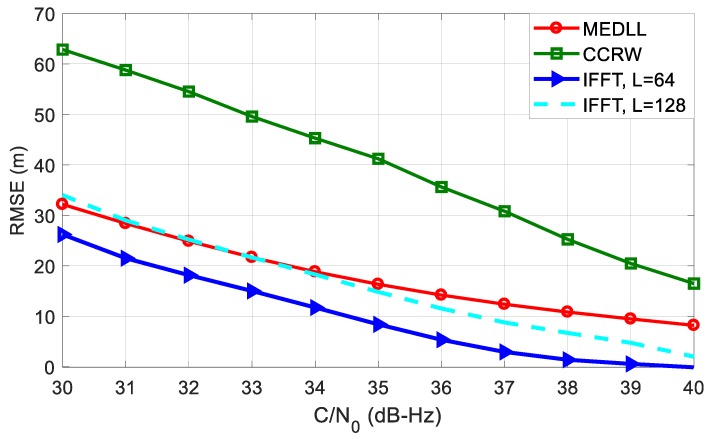
RMSE curves of different methods for BOC(14,2) signal with single reflected path.

Figure 14 shows the circumstance when the incoming signal is a BOC(14,2) modulated signal. The code rate of BOC(14,2) is 2.046 MHz. Because the time delay of the reflected ray is still 75 metres, equivalent to 0.51 code chip, the correlation peak of multipath signal shifts significantly to the right. Figure 15 indicates the performance of code delay estimation in the case of BOC(14,2). We can see that the CCRW method has the worst performance among all methods. When C/N_0_ is 30 dB-Hz, the RMSE is more than 60 m. That is because high-order BOC signals have more correlation peaks compared with the low-order BOC signals. The CCRW methods try to suppress these secondary peaks, and meanwhile have to suffer a power loss of the main peak, which will cause significant ranging errors. So for this case, the CCRW methods cannot work well.

Interestingly, compared with the results in Figure 11, the RMSE curves of two aFFT methods even decrease slightly instead of going up. That’s probably because of the good anti-multipath capacity of BOC(14,2) signal, but for MEDLL, the multi-peaks of BOC(14,2) still cause a RMSE increase compared with the BOC(1,1) signal.

**Figure 16:**
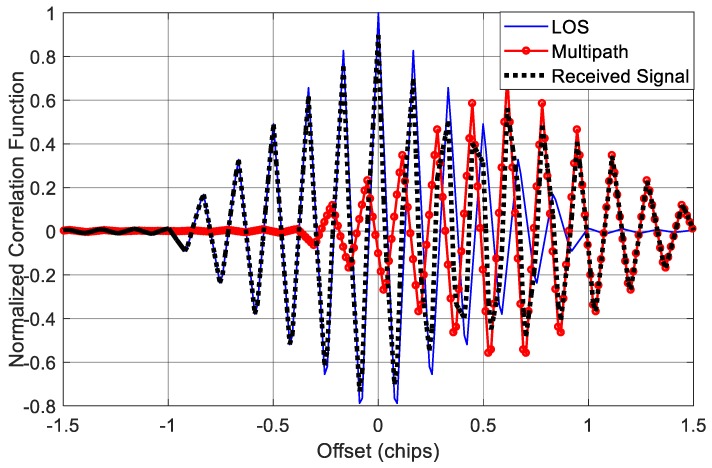
Correlation function of BOC(15,2.5) signal in the presence of single multipath component.

**Figure 17:**
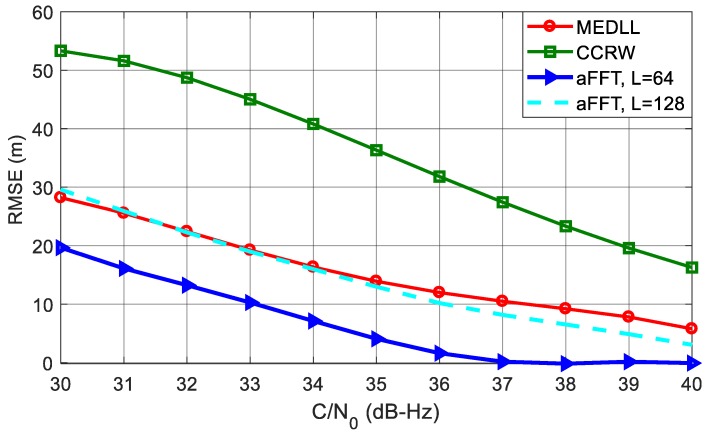
RMSE curves of different methods for BOC (15,2.5) signal with single reflected path.

Further, the correlation function and RMSE curves of BOC(15,2.5) are shown in Figure 16 and Figure 17, respectively. These results are quite similar to that of BOC(14,2). However, BOC(15,2.5) has a code rate of 2.5 × 1.023 MHz, which is larger than the code rate of BOC(14,2), so the RMSE curves of different approaches are slightly lower than the curves in Figure 15.

From all simulation results we present above, the proposed averaging-FFT method (*L* = 64) outperforms all other methods either in a single-path or multi-path channel and either for low-order or high-order BOC modulated signals. It means the proposed method has good universality and robustness. It is feasible to perform multipath parameter estimation for arbitrary type of BOC(*m*,*n*) signal with various numbers of reflected paths.

## 6. Conclusions

In this paper, we have developed a parametric method aiming at multipath estimation in the frequency domain for arbitrary BOC signals as well as BPSK signal. It takes advantage of the processes of segmentation and averaging to deal with the imperfections of conventional frequency domain technique—high computation complexity and vulnerability to the noise. These changes almost halve the computation amount and meanwhile significantly improve the feasibility and robustness.

The multipath suppression performance was well evaluated in three simulation scenes: (1) single-path channel; (2) three-path channel; and (3) high-order BOC signals. Results show that the number of reflected path has very limited effect on the proposed method, but significant impact on the CCRW and MEDLL method. When C/N_0_ is 40 dB-Hz, the RMSE of aFFT method (*L* = 64) is almost smaller than CCRW and MEDLL in a three-path channel by 60 m and 50 m, respectively. Besides, when the incoming signal is a high-order BOC signal, such as BOC(14,2) signal, for a C/N_0_ of 30 dB-Hz, the RMSE of the aFFT method (*L* = 64) is smaller than CCRW by 37 m. Thus, the proposed method has good robustness in severe multipath environments with lower computational load for both low-order and high-order BOC signals and its performance becomes better as the value of the window length *L* decreases.

## Figures and Tables

**Table 1:** Setting of Multipath Parameter Estimation.

Parameter	Value
Signal modulation type	BOC(1,1)
Signal length	40 ms
Sampling rate	20 MHz
Window length *L*	128 points
Window function	Hamming
Multipath attenuation (power)	−3 dB
C/N_0_	36 dB-Hz

**Table 2:** Setting of Multipath Parameter Estimation.

Parameter	Value
Signal modulation type	BPSK(1)
Signal length	20 ms
Sampling rate	25 MHz
Window length *L*	128 points
Window function	Hamming
C/N_0_	Larger than 45 dB-Hz

**Table 3:** Setting of Simulation Parameters.

Parameter	Value
Signal modulation type	BOC(1,1)
Signal length	40 ms
Sampling rate	20 MHz
Window length L	64, 128 points
Window function	Hamming
Multipath attenuation (power)	−3 dB
Multipath delay	75 m
